# Food literacy and its relationship with food intake: a comparison between adults and older adults using 2021 Seoul Food Survey data

**DOI:** 10.4178/epih.e2023062

**Published:** 2023-07-03

**Authors:** Seulgi Lee, Sohyun Park, Kirang Kim

**Affiliations:** 1 Department of Food Science and Nutrition, Dankook University, Cheonan, Korea; 2 Department of Food Science and Nutrition, Hallym University, Chuncheon, Korea; 3 The Korean Institute of Nutrition, Hallym University, Chuncheon, Korea

**Keywords:** Food literacy, Vegetables, Fruit, Adult, Aged

## Abstract

**OBJECTIVES:**

This study was conducted to examine the differences in food literacy between adults and older adults and the association of food literacy with food group intake.

**METHODS:**

In total, 4,039 participants from the 2021 Survey for Food Consumption in Seoul were included in this study. The intake of whole grains, high-protein foods, total vegetables, vegetables excluding kimchi and pickles, fresh fruits, and milk and dairy products was assessed using a simple food frequency questionnaire. Food literacy was measured using a food literacy measurement questionnaire.

**RESULTS:**

With the exception of milk and dairy, adults had a significantly higher proportion of insufficient food intake than older adults (p<0.001). Both adults and older adults with sufficient food group consumption had higher food literacy scores. Notably, the difference in food literacy scores by level of food intake was highest for fresh fruits (p<0.001). After adjusting for confounding factors affecting food literacy scores, a higher score was associated with a lower probability of having insufficient food intake in all food groups for both adults and older adults. Specifically, those in the highest food literacy score quartile were less likely to have insufficient intake of vegetables (odds ratio [OR], 0.35; 95% confidence interval [CI], 0.28 to 0.43) and fresh fruits (OR, 0.32; 95% CI, 0.24 to 0.43), compared to the lowest quartile.

**CONCLUSIONS:**

Improving food literacy is an important factor for promoting healthy food intake in older adults as well as adults. Therefore, it is necessary to develop intervention programs to work toward this goal.

## GRAPHICAL ABSTRACT

[Fig f1-epih-45-e2023062]

## INTRODUCTION

In Korea, the population of older adults aged 65 years and above constituted 17.5% of the total population in 2022, and this figure is expected to rise to 25.5% by 2030, resulting in an ultra-aged society. Consequently, the number of older adults with chronic diseases is also gradually increasing [1]. At the Primary Health Care Conference, held to commemorate the 40th anniversary of the Alma Ata Declaration, the World Health Organization emphasized the importance of strengthening and supporting individuals’ capacity to acquire the knowledge, skills, and resources necessary for maintaining their own health and care [2]. In this context, health literacy has emerged as an important factor in managing and promoting health issues in daily life. Health literacy refers to the cognitive and social skills that determine a person’s motivation and ability to access, understand, and use health-related information [3,4]. Studies have shown that individuals with low health literacy possess less knowledge about chronic diseases [5], have a higher prevalence of chronic diseases, and exhibit insufficient ability to manage their health in daily life [6]. Additionally, a decline in cognitive function among older adults has been reported [7]. Health management abilities encompass the capacity to select and consume healthy foods in a rapidly changing dietary environment. As such, food literacy, which pertains to the ability to accurately understand and assess information related to diet and nutrition, is important for preventing geriatric diseases.

Recently, research on food literacy in the context of food and nutritional intake has been actively conducted outside of Korea, stemming from the concept of health literacy [8-11]. Food literacy is defined as the capacity to make informed decisions for health promotion and a sustainable food system, considering various factors; it also includes the ability to develop a positive relationship with food throughout one’s lifetime, in conjunction with food technology and behavior in the complex food system [12]. The key domains of food literacy include functional, interactive, and critical areas. The functional domain encompasses food-related knowledge and skills, self-efficacy and confidence, and dietary behaviors. The interactive domain involves cultural aspects of food intake and choice within the community, as well as fostering positive relationships with food and sharing with others. The critical domain includes recognizing the impact of food decisions on society and the environment, as well as considering societal and environmental sustainability [13].

Previous studies have reported that low food literacy scores are associated with nutritional imbalances and reduced meal diversity [14]. In Korea, food literacy measures for adults have recently been developed [15,16]. Yoo et al. [16] introduced a comprehensive concept of food literacy and created a food literacy questionnaire for adults that assesses 3 main areas: nutritional and food safety literacy (functional), cultural and relationship food literacy (interactive), and socio-ecological food literacy (critical). However, these 3 domains of food literacy may be influenced differently depending on the target population, making it necessary to consider various contextual factors on the individual level [13,17].

In health literacy research, Korean studies have primarily focused on older adults, finding that their health literacy levels are low relative to other age groups [18]. Consequently, it is essential to conduct separate studies on the factors influencing food literacy in older adults, who are at risk of physical and cognitive decline. However, in Korea, the conceptual understanding and measurement of food literacy in older adults is lacking, making it challenging to determine whether food literacy measures are appropriate for this age group. Therefore, this study was conducted to investigate the differences in food literacy between adults and older adults, as well as to analyze the relationship between food literacy and the sufficiency of food intake to determine the feasibility of measuring food literacy among older adults.

## MATERIALS AND METHODS

### Data and participants

The study involved 4,039 adult participants aged 18 years or older who took part in the 2021 Seoul Food Survey, conducted from September 13, 2021 to October 29, 2021. These respondents were selected from 2,000 households in Seoul using stratified cluster sampling.

### Socio-demographic factors

The demographic and sociological characteristics of the participants included gender, age, educational background, household type, average monthly household income, and food security. Age was divided into 2 categories: adults aged 18 to 64 and older adults aged 65 or older. Educational background was separated into 3 groups: less than middle school graduate, high school graduate, and college entrant or higher. Households were classified into 4 groups: single-person households, couple households, households with 2 or more people, and other households. Average monthly household income was categorized as less than 2.0 million Korean won (KRW), between 2.0 million KRW and 3.5 million KRW, between 3.5 million KRW and 5.0 million KRW, and more than 5.0 million KRW. Food security levels were assessed using the food security questionnaire from the Korean National Health and Nutrition Examination Survey (KNHANES) [19]. Food security levels were classified as “sufficient quantity and variety of food,” “sufficient quantity of food, but not always a variety of food,” and “insufficient quantity and variety of food.”

### Frequency and sufficiency of food group intake

Food group intake was assessed using a simple food group intake frequency questionnaire, which was designed to analyze the adequacy of daily food group consumption among adults and older adults in Seoul. The food groups incorporated in the questionnaire included whole grains, high-protein foods (such as roasted, deep-fried, stewed, and souped meat, processed meat, fish, eggs, beans, and soybean products), vegetables (raw vegetables, vegetable side dishes, kimchi, and pickles), fresh fruits, and milk and dairy products. The frequency of intake was measured on a 9-point scale, ranging from less than once a month to more than 3 times a day (specifically: less than once a month, once a month, 2-3 times a month, 1-2 times a week, 3-4 times a week, 5-6 times a week, once a day, twice a day, and more than 3 times a day).

The sufficiency of food group intake was analyzed using the Korean Health Eating Index criteria by gender and age from the KNHANES [20]. The intake frequency criteria for each food group were as follows: whole grains, at least 0.3 times per day for all participants; high-protein foods, at least 5.0 times per day for men, 4.0 times per day for women and older men, and 2.5 times per day for older women; total vegetables, at least 8.0 times per day for men, women, and older men and 6.0 times per day for older women; vegetable intake excluding kimchi and pickled vegetables, at least 5.0 times per day for men, women, and older men and 3.0 times per day for older women; fresh fruits, at least 1.5 times per day for men, 1.0 time per day for women and older men, and 0.5 times per day for older women; and milk and dairy products, at least 1.0 time per day for all participants.

### Food literacy

Food literacy was assessed using a previously developed questionnaire for adults [16]. The questionnaire items were categorized into 3 domains: nutrition and safety, cultural and relational, and socio-ecological. Within the nutrition and safety domain, the maximum score was 70 points, based on a 5-point scale with 14 items. The cultural and relational domain had a maximum score of 40 points, using a 5-point scale with 8 items. Lastly, the socio-ecological domain was evaluated with a maximum score of 55 points, consisting of 11 items on a 5-point scale. Each domain was standardized to a 100-point scale, and the sum of the 3 domains was divided by 3 to calculate the average food literacy score.

### Statistical analysis

All statistical analyses were weighted based on individual sampling weights, which were generated to account for the complex sample design and to calculate representative values for current Seoul citizens. The comparison of socio-demographic factors and the sufficiency of food group intake between adults and older adults was conducted using the chi-square test and presented as frequency (n) and percentage (%). The comparison of food literacy scores by age group and sufficiency of food group intake was performed using the t-test. To determine the probability of having insufficient food group intake based on food literacy score, logistic regression analysis was conducted, adjusting for confounding variables that may affect the sufficiency of food group intake. All analyses were performed using SPSS version 26.0 (IBM Corp., Armonk, NY, USA), and significance testing was conducted at the level of α< 0.05.

### Ethics statement

The study protocol received approval from the Institutional Review Board of Dankook University (IRB No. 2021-07-052-003), and informed consent was obtained from all participants.

## RESULTS

### General characteristics of participants

Of the 4,039 participants, 3,395 (84.1%) were adults and 644 (15.9%) were older adults. The participants included 1,943 men (48.1%) and 2,096 women (51.9%). The average age of the adults was 42.2 years, while the older adults had an average age of 70.4 years. Most adults had a college education or higher (70.9%), whereas the majority of older adults had only completed middle school or below (48.0%). Regarding household types, 64.1% of adults belonged to households with 2 or more members, which accounted for the largest proportion. Among older adults, 52.1% were part of couple households, followed by 34.1% in single-person households. The average monthly household income for adults was predominantly above 5.0 million KRW (47.1%), while 43.5% of older adults had an income of less than 2.0 million KRW. The percentage of individuals who did not have sufficient food was 17.5% among adults and 35.2% among older adults. The proportion of older adults experiencing food insufficiency was higher than that of the adult group (Table 1).

### Sufficiency of food group intake in adults and older adults

Among all participants, whole grains had the highest proportion of sufficient intake at 61.8%, followed by vegetables excluding kimchi and pickles (45.6%). The food group with the highest proportion of insufficient intake was high-protein foods at 87.7%. Similar patterns were observed in adults and older adults, and the proportion of insufficient intake was significantly higher in adults than older adults for all food groups except for milk and dairy products (p<0.001; Table 2).

### Food literacy in adults and older adults by item

Table 3 displays the food literacy scores for both adults and older adults. The overall average food literacy score was 61.4 points, with adults scoring significantly higher than older adults (61.6 vs. 60.5, p=0.007). Specifically, adults had higher scores than older adults (59.9 vs. 57.9) in the relationship and culture domain. Overall, the social and ecological domain had the highest mean score (62.7), followed by the nutrition and safety domain (62.0) and the relationship and culture domain (59.6). Both adults and older adults exhibited similar patterns across these domains.

Upon comparing the questions within each domain, we discovered that in the nutrition and safety domain, adults scored higher on questions related to knowledge (Q3, Q4, Q5, Q6, and Q13), while older adults scored higher on questions related to practice (Q9, Q11, and Q12). In the relationship and culture domain, adults had higher scores on questions related to enjoying various cultures and cooking (Q15, Q17, and Q21), while older adults scored higher on questions related to relationships (Q18 and Q19). Within the social and ecological domain, adults had higher scores on questions related to the environment and ecology (Q28, Q29, and Q32), while older adults’ scores were significantly higher on questions related to agriculture and production (Q23, Q24, Q25, Q26, Q27, and Q31).

### Food literacy scores by sufficiency of food group intake

The comparison of food literacy scores based on the sufficiency of food group intake is presented in Table 4. In all domains, participants with sufficient food group consumption had significantly higher food literacy scores relative to those with insufficient consumption. This pattern was observed in both adult and older adult populations.

Specifically, regarding fresh fruits, the food literacy score for the entire participant pool was 66.9 points for the group with sufficient fresh fruit intake and 61.5 points for the insufficient intake group, demonstrating the largest difference among food groups. The difference was 6.2 points for adults and 5.0 points for older adults (p<0.001). When analyzing food literacy scores by domain, we observed a significant difference in the nutritional and safety domain scores, particularly in relation to the sufficiency of fresh fruit intake. This difference was 10.3 points for all participants, 10.9 points for adults, and 11.9 points for older adults (p<0.001).

### Relationship between insufficient food group intake and food literacy

Table 5 presents the results of the analysis examining the relationship between inadequate food group intake and food literacy level, after adjusting for confounding factors significantly impacting food literacy score. In all food groups, a higher food literacy score was associated with a lower odds ratio (OR) for insufficient intake of the food group, and this was consistent for both adults and older adults. Overall, among the food groups, the likelihood of having insufficient intake was lowest for fresh fruit (OR, 0.96; 95% confidence interval [CI], 0.95 to 0.97). Notably, adults and older adults with higher food literacy scores were less likely to have insufficient total vegetable (OR, 0.96; 95% CI, 0.95 to 0.97) and insufficient fresh fruit (OR, 0.92; 95% CI, 0.89 to 0.95) intakes, respectively.

When the food literacy scores were divided into quartiles, the likelihood of insufficient intake in all food groups decreased as the quartile increased, relative to Q1. The food groups that demonstrated a clear trend of decreasing probability of insufficient intake were total vegetables and fresh fruits. For fresh fruits, the likelihood of insufficient intake decreased by approximately 76% for adults and approximately 85% for older adults in Q4 compared to Q1.

## DISCUSSION

Health literacy among older adults has been the subject of extensive investigation, while research on food literacy, a concept derived from health literacy, has been relatively limited. This study aimed to explore potential differences in food literacy scores between adults and older adults, as well as to examine the positive impact of food literacy on food intake among older adults.

In this study, the participants were Seoul residents who participated in the 2021 Seoul Food Survey, with 64.8% of older adult participants deemed to have sufficient quantity and quality regarding food security. Among the food groups, older adults exhibited the most insufficient consumption of milk and dairy products, followed by high-protein foods. The food groups with the greatest difference in insufficient intake between adults and older adults were vegetables and fresh fruits. This finding aligns with the results of the KNHANES, where the largest difference between the 2 groups was observed in total vegetable and fruit consumption, with older adults exhibiting a higher intake than adults. According to the Korea Health Statistics 2021, the difference between the 2 groups was 31.5 g for fruits and 46.6 g for total vegetables, including vegetables, mushrooms, and seaweed [21].

The total food literacy score differed between adults and older adults, with higher scores observed in adults. Upon examining the 3 domains of food literacy, the nutrition and safety domain, which assesses knowledge about food and nutrition as well as the ability to comprehend and utilize information about ingredients and cooking, revealed that knowledge-related questions received higher scores in adults, while older adults scored higher on ability-related questions. This domain displayed the largest difference in scores based on the adequacy of fresh fruit intake, aligning with Spronk et al. [22]’s 2014 finding that an increase in nutrition-related knowledge and practice corresponds to increased fresh fruit consumption.

The relationship and cultural domain encompasses an individual’s interest in and understanding of food culture, ability to promote well-being through food, the pursuit of enjoyment and meaning through food, and gastronomic interest in food. Within this domain, social network-related aspects, such as enjoying cooking, eating, or sharing food with family, friends, and neighbors, or engaging in conversations about food with those around them, can heavily influence the dietary intake of older adults. Previous studies have shown that living alone can lead to a lack of motivation for cooking, which may negatively impact healthy eating habits, resulting in decreased appetite and ultimately reduced food intake [23,24]. Moreover, several cohort studies have reported that older adults who are widowed, living alone, or have infrequent social contact exhibit poor dietary quality, including lower fruit and vegetable variety [25,26]. In line with these findings, our study revealed that the scores for certain relationship-related questions on food literacy were higher for older adults compared to younger adults. Furthermore, the scores in this domain for older adults exhibited a notable difference based on the sufficiency of intake for most food groups.

There is a growing global trend of increased interest in healthy diets and sustainable foods, along with a positive perception of sustainable food [27]. In this study, the social and ecological domain received the highest score among the various domains of food literacy. This domain encompasses the ability to understand and evaluate the diverse social and ecological consequences associated with individual food choices, reflecting one’s interest in and perception of food-related inequality and coexistence. Among the questions, adults scored higher than older adults in areas related to the environment and ecology, such as animal welfare, fair trade, and food packaging. Conversely, older adults achieved higher scores in questions related to agriculture and production. Notably, older adults placed high importance on seasonal foods and environmentally friendly agricultural products.

Food literacy, which encompasses individual behaviors in planning, selecting, preparing, and consuming healthy foods, is essential for fostering sustainable eating habits and improving well-being [17]. Previous studies have demonstrated a positive association between food literacy and food security, health indicators, and healthy food intake [28-32]. Several recent studies have also found a connection between food literacy and obesity, suggesting that food literacy plays an important role in making healthy food choices and maintaining behavior changes to support a healthy weight [30,32]. A study focusing on the Italian population discovered that limited food literacy was more common among older adults, particularly those with lower education levels and financial difficulties [30].

In the present study, the likelihood of insufficient consumption decreased across all food groups as food literacy scores increased, even after adjusting for confounding factors. Specifically, the probabilities of insufficient fresh fruit intake in all participants, total vegetable intake in adults, and fresh fruit intake in older adults were significantly reduced. Considering the results of a previous study, which demonstrated that an education program aimed at improving food literacy significantly increased the consumption of fresh fruits and vegetables among adults and older adults [33], food literacy can be considered an important dietary indicator reflecting healthy food intake levels for adults in general and older adults in particular.

This study had several limitations. First, the survey participants were adults and older adults living in a large city, which may yield different results from adults and older adults residing in rural areas. Previous studies have demonstrated that the food environment is significantly associated with older adults’ vegetable and fresh fruit intake in both urban and rural areas, suggesting that the impact of food literacy on food intake may vary between regions [34,35]. Therefore, further research targeting older adults in rural areas is necessary. Second, this study determined the sufficiency of food group intake using the Korean Health Eating Index criteria, which were developed based on serving sizes for the food groups. However, since dietary intake was assessed using a simple food frequency questionnaire with frequency responses in the present study, sufficiency was evaluated using intake frequency rather than serving size. This approach has the potential to overestimate dietary intake [36]. Nonetheless, the inclusion of portion sizes in the food frequency questionnaire remains controversial, as portion sizes are often poorly estimated [37]. Previous studies have reported that a large percentage of between-person variation can be explained by intake frequency rather than portion size, indicating that information regarding a single serving size is already accounted for by intake frequency [36]. Despite this, it may be necessary to confirm the results of evaluating intake sufficiency based on nutrient intake calculated by including serving sizes in the food frequency questionnaire. Lastly, a limitation exists in determining whether the difference in food literacy measurements between older adults and adults results from an actual difference between these groups or a bias in the measurement tool for older adults, as the validity of the food literacy measurement tool has not been studied for that population. Consequently, it is essential to verify the tool’s validity for older adults. Although the validity was limited, this study confirmed the feasibility of measuring food literacy in older adults by demonstrating a positive relationship with healthy food intake and results consistent with adults. In future studies, additional qualitative investigations on item recognition are needed for socially and economically vulnerable older adults, as food literacy questions are lengthy and may contain unfamiliar terms.

Although the study of Korean food literacy is still in its early stages and primarily focuses on adults, our research revealed a positive correlation between food literacy and food group consumption among urban older adults, utilizing data from the 2021 Seoul Food Survey. Consequently, enhancing food literacy in older adults may play a crucial role in encouraging healthy food intake, making it essential to develop programs targeting this population.

## Figures and Tables

**Figure f1-epih-45-e2023062:**
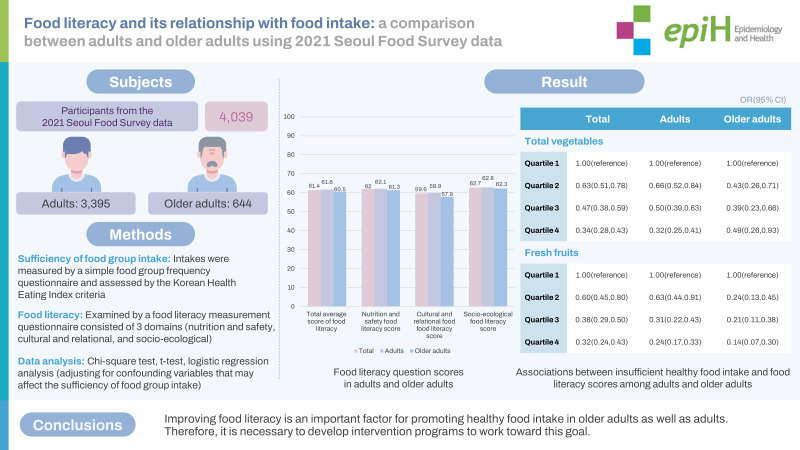


**Table 1.** General characteristics of participants in adult and older adult populations

Characteristics		Total	Adults	Older adults	p-value
Gender		4,039 (100)	3,395 (84.1)	644 (15.9)	0.271
	Men	1,943 (48.1)	1,646 (48.5)	297 (46.1)	
	Women	2,096 (51.9)	1,749 (51.5)	347 (53.9)	
Age, mean±SD (yr)		46.6±16.1	42.2±13.3	70.4±5.1	<0.001
Education level					<0.001
	≤Middle school	404 (10.0)	95 (2.8)	309 (48.0)	
	High school	1,173 (29.0)	892 (26.3)	281 (43.6)	
	≥College	2,462 (61.0)	2,408 (70.9)	54 (8.4)	
Household type					<0.001
	Single	724 (17.9)	505 (14.9)	219 (34.1)	
	One-generation	967 (23.9)	632 (18.6)	335 (52.1)	
	Two-generation or more	2,263 (56.0)	2,176 (64.1)	87 (13.5)	
	Other	85 (2.1)	83 (2.4)	2 (0.3)	
Household income (million KRW)					<0.001
	≤2.0	389 (9.6)	109 (3.2)	280 (43.5)	
	2.0 to 3.5	931 (23.0)	708 (20.8)	223 (34.6)	
	3.5 to 5.0	1,047 (25.9)	981 (28.9)	66 (10.2)	
	>5.0	1,673 (41.4)	1,598 (47.1)	75 (11.6)	
Food security					<0.001
	Sufficient food quantity & variety	3,208 (79.7)	2,792 (82.5)	416 (64.8)	
	Sufficient food quantity & insufficient variety	642 (16.0)	463 (13.7)	179 (27.9)	
	Insufficient food quantity & variety	175 (4.3)	128 (3.8)	47 (7.3)	

Values are presented as number (%). SD, standard deviation; KRW, Korean won.

**Table 2.** Food group intake in adults and older adults

Components^1^		Total	Adults	Older adults	p-value
Whole grains					<0.001
	Sufficient	2,496 (61.8)	1,991 (58.6)	506 (78.6)	
	Insufficient	1,543 (38.2)	1,404 (41.4)	138 (21.4)	
Meat, fish, eggs, beans					<0.001
	Sufficient	497 (12.3)	334 (9.8)	164 (25.5)	
	Insufficient	3,542 (87.7)	3,062 (90.2)	480 (74.5)	
Total vegetables					<0.001
	Sufficient	1,436 (35.6)	1,040 (30.6)	396 (61.6)	
	Insufficient	2,603 (64.4)	2,355 (69.4)	247 (38.4)	
Vegetables excluding kimchi and pickles					<0.001
	Sufficient	1,842 (45.6)	1,359 (40.0)	483 (75.0)	
	Insufficient	2,197 (54.4)	2,037 (60.0)	161 (25.0)	
Fresh fruits					<0.001
	Sufficient	949 (23.5)	642 (18.9)	307 (47.7)	
	Insufficient	3,090 (76.5)	2,753 (81.1)	337 (52.3)	
Milk and dairy products					0.253
	Sufficient	716 (17.7)	612 (18.0)	104 (14.5)	
	Insufficient	3,323 (82.3)	2,783 (82.0)	540 (83.9)	

Values are presented as number (%).

1 Susfficient was defined as meeting or exceeding the intake frequency criterion for each food group.

**Table 3.** Food literacy question scores in adults and older adults

Food literacy questions		Total	Adults	Older adults	p-value
Total average score of food literacy		61.4±10.0	61.6±10.2	60.5±9.2	0.007
Nutrition and safety food literacy score		62.0±13.0	62.1±13.1	61.3±12.3	0.105
	Q1. I know about the various food groups that make up nutritionally balanced meals	3.4±0.8	3.4±0.8	3.4±0.8	0.922
	Q2. I try to eat a variety of food groups such as cereals, fish, vegetables, fruits, and dairy	3.5±0.8	3.5±0.8	3.5±0.8	0.065
	Q3. I make a list of items I need to buy before grocery shopping	3.3±0.9	3.3±0.8	3.2±0.9	0.002
	Q4. I can understand the food labeling in packages of processed foods	3.4±0.8	3.4±0.8	3.2±0.8	<0.001
	Q5. When purchasing processed food, I check food information (ingredients, nutrition facts)	3.2±0.9	3.3±0.9	3.1±0.8	<0.001
	Q6. I check the country of origin when purchasing food	3.4±0.9	3.4±0.9	3.3±0.8	0.012
	Q7. I know how to separate and store ingredients that I cannot consume immediately	3.5±0.8	3.5±0.8	3.5±0.7	0.507
	Q8. I can follow a simple recipe	3.5±0.9	3.5±0.9	3.5±0.9	0.816
	Q9. I can prepare a meal without difficulty	3.4±1.0	3.4±1.0	3.7±1.0	<0.001
	Q10. I wash my hands thoroughly before cooking	4.2±0.7	4.2±0.7	4.2±0.8	0.421
	Q11. I know how to store food in the refrigerator and understand impacts of room-temperature storage on freshness and safety	3.4±0.9	3.4±0.9	3.5±0.8	0.010
	Q12. I check the cleanliness of restaurants when eating out	3.5±0.9	3.5±0.9	3.6±0.8	0.026
	Q13. I can understand information related to food safety issues in the medi	3.4±0.8	3.4±0.8	3.2±0.7	<0.001
	Q14. I can critically evaluate food advertisement content, especially health claims	3.4±0.7	3.4±0.7	3.4±0.7	0.004
Cultural and relational food literacy score		59.6±12.0	59.9±12.1	57.9±11.3	<0.001
	Q15. Cooking is enjoyable	3.2±0.8	3.2±0.8	3.0±0.8	<0.001
	Q16. When eating, I fully concentrate on eating	3.4±0.8	3.4±0.8	3.4±0.7	0.171
	Q17. When eating, I savor various senses such as visual beauty, aroma, taste, and texture	3.4±0.8	3.5±0.8	3.3±0.8	<0.001
	Q18. I am grateful for the process that has allowed the food to arrive at the table	3.5±0.8	3.5±0.8	3.6±0.8	0.031
	Q19. I like to eat or share food with my family, friends, and neighbors	3.4±0.8	3.4±0.8	3.5±0.8	0.019
	Q20. I enjoy talking about food with people around me	3.3±0.8	3.3±0.8	3.4±0.9	0.193
	Q21. I am interested in food from various cultures	3.2±0.8	3.3±0.8	2.8±0.8	<0.001
	Q22. Enjoying traditional food can help protect our cultural identity	3.5±0.8	3.5±0.8	3.5±0.7	0.044
Socio-ecological food literacy score		62.7±10.1	62.8±10.2	62.3±9.4	0.246
	Q23. I know why choosing seasonal food is good for the environment	3.7±0.8	3.6±0.8	3.8±0.9	<0.001
	Q24. I think food that is directly traded with producers is more reliable	3.5±0.8	3.5±0.8	3.6±0.8	0.004
	Q25. I think choosing organic products is important for environmental conservation	3.7±0.8	3.7±0.8	3.8±0.8	0.008
	Q26. I think rural farmers are important for a sustainable society	3.6±0.7	3.6±0.7	3.7±0.6	0.048
	Q27. I am interested in urban agriculture (such as city gardening, weekend farming, etc.)	3.0±1.0	3.0±1.0	3.2±1.0	<0.001
	Q28. It is important to consider animal welfare when purchasing meat and eggs	3.4±0.8	3.5±0.8	3.1±0.8	<0.001
	Q29. I know why it is better to choose fair-trade products	3.3±0.8	3.4±0.8	3.1±0.8	<0.001
	Q30. I believe that reducing meat and promoting vegetarianism helps slow climate change	3.7±0.7	3.7±0.7	3.7±0.7	0.607
	Q31. I try to reduce food waste	3.4±0.8	3.4±0.8	3.5±0.8	0.033
	Q32. I try to reduce food packaging waste (take-out drinks, delivery foods, etc.)	3.2±0.9	3.3±0.9	3.1±0.9	<0.001
	Q33. I think everyone should have access to quality food regardless of economic circumstances	3.9±0.6	3.9±0.6	3.9±0.6	0.101

Values are presented as mean±standard deviation.

**Table 4.** Food literacy scores by sufficiency of food group intake

Components^1^		Total				Adults				Older adults			
		Total	Nutrition and safety	Cultural and relational	Socioecological	Total	Nutrition and safety	Cultural and relational	Socioecological	Total	Nutrition and safety	Cultural and relational	Socioecological
Total		61.4±10.0	62.0±13.0	59.6±12.0	62.7±10.1	61.6±10.2	62.1± 13.1	59.9±12.1	62.8±10.2	60.5±9.2	61.3±12.3	57.9±11.3	62.3±9.4
Whole grains													
	Sufficient	62.1±9.8	63.4±12.9	60.0±11.6	63.0±9.8	62.4±10.0	63.8±13.0	60.4±11.8	63.1±9.9	61.0±9.0	61.9±12.1	58.7±10.9	62.4±9.5
	Insufficient	60.2±10.3	59.6±12.9	58.8±12.6	62.3±10.4	60.4±10.3	59.7±12.9	59.2±12.6	62.3±10.6	58.6±10.0	58.7±12.8	55.0±12.6	62.0±9.0
	p-value	<0.001	0.002	0.035	<0.001	<0.001	<0.001	0.005	0.024	0.006	0.006	0.001	0.662
Meat, fish, eggs, beans													
	Sufficient	64.9±8.2	66.9±10.0	62.8±10.7	64.9±8.7	65.2±8.6	67.2±10.4	63.6±10.7	64.8±9.2	64.2±7.3	66.2±9.1	61.3±10.4	65.0±7.6
	Insufficient	60.9±10.2	61.3±13.2	59.1±12.1	62.4±10.2	61.2±10.3	61.6±13.3	59.5±12.2	62.6±10.3	59.3±9.5	59.6±12.8	56.8±11.4	61.4±9.8
	p-value	<0.001	<0.001	<0.001	<0.001	<0.001	<0.001	<0.001	<0.001	<0.001	<0.001	<0.001	<0.001
Total vegetables													
	Sufficient	64.0±8.8	65.2±12.6	61.3±10.7	65.5±9.4	64.8±8.9	66.1±13.0	62.1±10.7	66.3±9.4	62.0±8.0	63.0±11.1	59.4±10.3	63.4±9.1
	Insufficient	60.0±10.4	60.2±12.9	58.6±12.6	61.2±10.1	60.2±10.4	60.4±12.8	58.9±12.6	61.3±10.1	58.2±10.5	58.5±13.6	55.5±12.5	60.5±9.6
	p-value	<0.001	<0.001	<0.001	<0.001	<0.001	<0.001	<0.001	<0.001	<0.001	<0.001	<0.001	<0.001
Vegetables excluding kimchi and pickles													
	Sufficient	63.4±8.9	64.4±12.5	61.0±10.8	64.7±9.4	64.0±9.0	65.1±12.8	61.7±10.8	65.2±9.5	61.6±8.1	62.7±11.4	59.1±10.5	63.2±8.9
	Insufficient	59.8±10.7	59.9±13.1	58.3±12.8	61.1±10.3	60.0±10.6	60.2±13.0	58.7±12.7	61.2±10.3	57.0±11.3	57.0±13.8	54.4±13.1	59.8±10.5
	p-value	<0.001	<0.001	<0.001	<0.001	<0.001	<0.001	<0.001	<0.001	<0.001	<0.001	<0.001	<0.001
Fresh fruits																									
	Sufficient	66.9±9.3	78.8±11.1	68.7±9.5	62.6±10.6	67.8±9.6	79.9±11.5	69.7±9.7	62.9±10.7	64.9±8.3	76.4±10.1	66.4±8.6	62.0±10.3
	Insufficient	61.5±9.9	68.5±15.5	59.9±13.2	58.6±12.3	61.6±10.0	69.0±15.4	60.4±13.2	59.2±12.3	59.9±9.7	64.5±15.4	56.5±13.3	54.3±11.0
	p-value	<0.001	<0.001	<0.001	<0.001	<0.001	<0.001	<0.001	<0.001	<0.001	<0.001	<0.001	<0.001
Milk and dairy products													
	Sufficient	65.4±7.9	67.2±10.3	61.5±10.6	67.4±9.2	63.2±9.1	64.7±11.2	60.2±11.0	64.7±10.5	63.1±7.3	65.5±9.2	58.1±11.1	65.8±7.2
	Insufficient	61.0±10.2	61.4±13.3	59.5±12.2	62.3±10.0	61.2±10.4	61.6±13.4	59.8±12.3	62.4±10.1	60.0±9.5	60.4±12.7	57.9±11.4	61.6±9.6
	p-value	<0.001	<0.001	<0.001	<0.001	<0.001	<0.001	0.346	<0.001	<0.001	<0.001	0.847	<0.001

Values are presented as mean±standard deviation.

1 Susfficient was defined as meeting or exceeding the intake frequency criterion for each food group.

**Table 5.** Associations^1^ between insufficient healthy food intake and food literacy scores among adults and older adults^2^

Components	Total	Adults	Older adults
Whole grains			
Total	0.98 (0.98, 0.99)	0.98 (0.98, 0.99)	0.96 (0.94, 0.99)
Quartile 1	1.00 (reference)	1.00 (reference)	1.00 (reference)
Quartile 2	0.97 (0.81, 1.17)	0.95 (0.77, 1.16)	1.15 (0.67, 1.96)
Quartile 3	0.82 (0.68, 1.00)	0.80 (0.65, 0.98)	0.99 (0.55, 1.77)
Quartile 4	0.67 (0.55, 0.82)	0.66 (0.53, 0.82)	0.72 (0.36, 1.45)
Meat, fish, eggs, beans			
Total	0.97 (0.96, 0.99)	0.98 (0.96, 0.99)	0.96 (0.94, 0.99)
Quartile 1	1.00 (reference)	1.00 (reference)	1.00 (reference)
Quartile 2	1.00 (0.71, 1.40)	0.97 (0.66, 1.43)	1.15 (0.56, 2.37)
Quartile 3	0.59 (0.43, 0.80)	0.89 (0.60, 1.30)	0.22 (0.11, 0.41)
Quartile 4	0.58 (0.42, 0.81)	0.59 (0.41, 0.85)	0.53 (0.26, 1.10)
Total vegetables			
Total	0.96 (0.95, 0.97)	0.96 (0.95, 0.97)	0.97 (0.95, 0.99)
Quartile 1	1.00 (reference)	1.00 (reference)	1.00 (reference)
Quartile 2	0.63 (0.51, 0.78)	0.66 (0.52, 0.84)	0.43 (0.26, 0.71)
Quartile 3	0.47 (0.38, 0.59)	0.50 (0.39, 0.63)	0.39 (0.23, 0.66)
Quartile 4	0.35 (0.28, 0.43)	0.32 (0.25, 0.41)	0.49 (0.26, 0.93)
Vegetables excluding kimchi and pickles			
Total	0.97 (0.96, 0.98)	0.97 (0.96, 0.98)	0.97 (0.95, 0.99)
Quartile 1	1.00 (reference)	1.00 (reference)	1.00 (reference)
Quartile 2	0.80 (0.66, 0.98)	0.79 (0.64, 0.98)	0.71 (0.42, 1.19)
Quartile 3	0.69 (0.57, 0.85)	0.70 (0.56, 0.86)	0.71 (0.40, 1.26)
Quartile 4	0.50 (0.40, 0.61)	0.47 (0.38, 0.58)	0.57 (0.28, 1.18)
Fresh fruits			
Total	0.96 (0.95, 0.97)	0.96 (0.95, 0.97)	0.92 (0.89, 0.95)
Quartile 1	1.00 (reference)	1.00 (reference)	1.00 (reference)
Quartile 2	0.60 (0.45, 0.80)	0.63 (0.44, 0.91)	0.24 (0.13, 0.45)
Quartile 3	0.38 (0.29, 0.50)	0.31 (0.22, 0.43)	0.21 (0.11, 0.38)
Quartile 4	0.32 (0.24, 0.43)	0.24 (0.17, 0.33)	0.14 (0.07, 0.30)
Milk and dairy products			
Total	0.98 (0.97, 0.99)	0.98 (0.97, 0.99)	0.96 (0.94, 0.99)
Quartile 1	1.00 (reference)	1.00 (reference)	1.00 (reference)
Quartile 2	0.55 (0.43, 0.72)	0.39 (0.29, 0.52)	0.49 (0.25, 0.97)
Quartile 3	0.54 (0.42, 0.70)	0.45 (0.34, 0.60)	0.40 (0.20, 0.80)
Quartile 4	0.58 (0.45, 0.76)	0.39 (0.29, 0.53)	0.51 (0.23, 1.13)

Values are presented as odds ratio (95% confidence interval).

1 By logistic regression.

2 Adjusted for gender, age, education level, household type, household income, and food security.
